# Critical Review of Visual Models for Police Use of Force Decision-Making

**DOI:** 10.3390/vision5010006

**Published:** 2021-01-28

**Authors:** Paula M. Di Nota, Peter Stoyko, Jodie Jenkinson, Evelyn C. Boychuk, Judith P. Andersen

**Affiliations:** 1 Department of Psychology, University of Toronto Mississauga, Mississauga, ON L5L 1C6, Canada; paula.dinota@utoronto.ca (P.M.D.N.); ecboychu@gmail.com (E.C.B.); 2 Elanica Information Design, B108-1241 Kilborn Place, Ottawa, ON K1H 1A5, Canada; peter.stoyko@elanica.com; 3 Graduate Program in Biomedical Communications, University of Toronto Mississauga, Mississauga, ON L5L 1C6, Canada; j.jenkinson@utoronto.ca; 4 Institute of Medical Science, University of Toronto, Toronto, ON M5S 1A8, Canada

**Keywords:** visual models, information visualization, critical decision-making, police training, use of force, de-escalation

## Abstract

Recent calls for widespread police reform include re-examination of existing training and practice surrounding the use of force (UOF, e.g., verbal and non-verbal communication, physical tactics, firearms). Visual models representing police UOF decision-making are used for both police training and public communication. However, most models have not been empirically developed or assessed in either the applied police or vision science literatures, representing significant gaps in knowledge. The purpose of the current review is to provide a novel, relevant, and practical analysis of the visual components of three common police UOF decision-making model types (circular, cyclical, staircase). We begin with a critical evaluation of the visual features specific to each model type (i.e., shape), followed by critical reviews of common visual features, including colour, implied motion, text, and clarity. The insights provided by the current work afford scientists from visual disciplines a unique opportunity to contribute meaningfully to the improvement of existing police UOF practices, with the goal of promoting public and occupational safety. To this end, we conclude with evidence-based recommendations for designing visual models that effectively promote training of police and communication of police UOF decision-making to the public.

## 1. Introduction

Public outcry over police encounters that result in the use of force (UOF), especially lethal force, against unarmed citizens or individuals suffering a mental health crisis has spanned decades but recently reached a boiling point. As with many concepts and practices in policing, UOF is inconsistently defined and operationalized across agencies [1]. As defined in this paper, UOF encompasses discrete verbal and non-verbal communication (including de-escalation) and physical skills and tactics (i.e., arrest, holding, and hand-to-hand defensive maneuvers) to bring a situation under control. Further, depending on individual agency resources and policies, UOF also encompasses available force options, such as oleoresin capsicum (OC) spray (i.e., pepper spray); baton; canine response; non-lethal weapons, including conduced energy weapons (commonly referred to by the Taser brand name) and beanbag guns; and duty weapons or firearms [2]. Finally, we also define UOF as the cognitive processes related to perceiving (i.e., attending to), understanding, and evaluating the current situation (i.e., situational awareness, threat assessment) and decision-making to select the appropriate UOF option [3]. 

Beginning in the 1970s, North American police agencies have increasingly used visual models in training contexts to represent the various elements of UOF defined above. According to the Canadian Association of Chiefs of Police (CACP) [4], a graphical UOF framework model serves three primary goals:

- Facilitate officer and public understanding surrounding the reasons (“why”) and manner by which (“how”) an officer may or may not respond with the UOF.
- Aid police officer training in UOF, including “continuous critical assessment and evaluation of each situation” [4] (p. 3), and promote understanding of the variety of force options available to them so they can adequately respond to potentially violent encounters.
- Provide a valuable framework tool to facilitate articulating—but not justifying—police UOF encounters.

As reviewed by various police stakeholders [5,6,7], a number of current police UOF models do not meet the primary goals of their intended use as stated by the CACP [4]. The purpose of this paper is to bridge information across disciplines to fill a significant gap in knowledge surrounding the effectiveness of common graphical models used in training police UOF and communicating police UOF decision-making to the public. The lack of empirical research in this area also precludes any diverging hypotheses on the most effective model or type and has led to the development of numerous models that may not align with principles of information visualization discussed presently. Researchers in ecological psychology have recently appraised the effectiveness of various types of schematic diagrams in communicating relationships between organisms and their environments [8]. In a similar vein, the current paper provides a novel critical analysis of graphical features used to communicate police UOF decision-making in both training and public dissemination contexts. 

The purpose of this critical review is to evaluate the visual features of commonly used graphical models of police UOF decision-making. To clarify, the current work is not an appraisal or criticism of existing *training practices* surrounding police UOF decision-making. We acknowledge that the main contributors to the design of current police UOF models are practitioners with valuable lived experience, but they are not experts in graphic or information design. The current work provides a novel and necessary contribution to the development of effective tools that can be practically used in police UOF training and communicating police UOF decision-making to the public (CACP goal #1).

The current review identifies three main types of police UOF decision-making models based on their visual characteristics: *circular models* and their multiple subtypes, which are often used within a single graphic, such as rings, pies, donuts, and crystal configurations (e.g., the Ontario Use of Force Model); *cycle models*, containing shapes, such as boxes or circles, connected by arrows implying direction or motion (e.g., the British Columbia Crisis Intervention and De-escalation (BC-CID) Model); and the linear continuum models, or *staircase models* (e.g., the Las Vegas Model). Each model type is exemplified by a publicly available police UOF model, and their unique visual components are critically analyzed from the perspective of vision science and information visualization. Next, we critically review the visual features common to all three model types, including colour, implied motion, text, and clarity. We also identify and discuss several practical challenges to visually communicating police UOF decision-making, including accessibility, sizing and graphic format, and secondary documentation. Together with continued collaboration between research scientists and police educators, the insights provided by the current critical review can contribute meaningfully to the improvement of existing police practices to promote public and occupational safety. To this end, we conclude our review with recommendations for designing effective UOF decision-making models for police.

## 2. Circular Models: Ontario Use of Force Model

**Figure 1:**
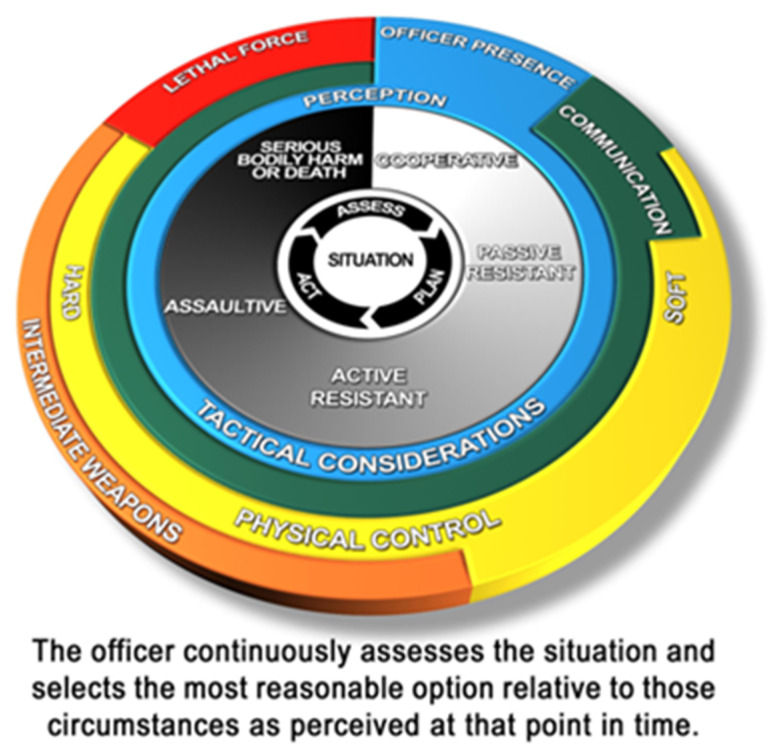
Ontario Use of Force Model. Image obtained from [6]. © Queen’s Printer for Ontario, 2004. Reproduced with permission.

Circular models are exemplified by the Ontario UOF Model (Figure 1). The national framework from which the current Ontario UOF Model was developed states that the model is intended to (a) assist in police UOF training and (b) serve “as a reference when making decisions and explaining their actions” [4] (p. 4). Recent reviews connecting police performance to stress physiology research suggest that it is very unlikely that highly complex models, like the Ontario UOF Model, would be consulted or recalled *during* a potential UOF encounter, unless deeply encoded into long-term implicit memory [3]. As a graphical form, the authors of the Ontario UOF Model rely on an implicit clock metaphor, implicit insofar as there are no overt cues to orient the viewer to that visual analogy. It is designed such that the viewer’s attention is first drawn to the twelve o’clock position and then proceeds to follow a clockwise sequence. Ideally, colour contrast (Section 5) and prominent shapes would attract the eye to that starting position, then progress through the force options and situational factors. In this case, however, visually dominant red and black backgrounds draw to the end of the sequence, likely causing momentary disorientation. Circular graphical formats have also been adopted with the intention of reflecting the non-linear nature of UOF encounters [6], such that forward progression (i.e., escalation) can also be dynamically reversed (i.e., de-escalation). The potential for this form to communicate non-linearity is undermined by other features, to be discussed in later sections.

### 2.1. Multiple Circular Chart Types in a Single Graphic

**Figure 2:**
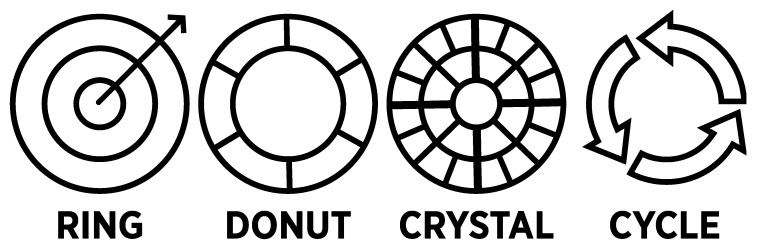
Circular chart types.

A major source of confusion with the Ontario UOF Model (Figure 1) is that it combines several different circular chart types that mostly work at cross-purposes (Figure 2). Although most of the chart types are familiar to many, there is nothing inherently self-evident about them—each had to be learned at some point. Most chart types (e.g., pie chart, line chart, bar graph) were invented by William Playfair in the mid-1800s [9] (pp. 18–19). Each was originally designed to display a certain type of information according to a coherent set of visual rules and conventions designed to facilitate interpretation. By layering different chart types, much of that coherence is lost. The observer does not know which rules and conventions apply, nor how conflicts are resolved. Therefore, the combination of multiple chart types is not recommended as it may lead to the conflation of information that is intended for communication through each distinct chart type [10,11,12].

**Ring charts** present a centre-to-edge dimension of hierarchically organized information. Central information is presented at the core, and additional layers radiate outward. One of the limitations of the Ontario UOF Model is that there is no way to tell what the center-to-edge dimension is. There is no thematic nor logical ordering to the layering of rings. Moreover, some cells in the chart cut across multiple rings, with no indication of what that means. Relations and ordering of rings may be taught to the police separately during training (see Section 9.3—Secondary Documentation), but as a public communication tool, it is unclear what movement from the centre to the outer rings (or vice versa) indicates, other than to grant visual prominence to the items at the centre. Occluded relationships between visual features are an example of the knowledge trap (see Section 7.2) [13]: experts become so familiar with an area of knowledge that they overlook the fact that certain features of the graphic are not self-evident, widely known, or easily interpreted by others. Interpretation may be harder for members of the lay public, who likely have little acquaintance with concepts related to police UOF decision-making in the first place.

Donut and pie charts categorize information into discrete segments. The size of the segments may suggest a quantitative value or signal that certain segments are of greater importance. As a quantitative chart, there is some debate about whether viewers can judge the relative sizes of segments accurately [9,14,15,16,17]. In the case of the Ontario UOF Model, physical control is represented by a much longer bar than lethal force but is considerably thinner in some parts. It is unclear why. In an independent review [7], the Honourable Justice Iacobucci also argued that the circular Ontario UOF Model de-emphasizes the role of communication and de-escalation (which does not appear at all on the model) in police UOF decision-making. The appropriateness of certain activities may be misaligned to the amount of space (hence visual prominence) provided in the chart, providing a misleading impression (see Section 8.2–Proportionality). Viewers also have somewhat visceral reactions to the shape of circular charts. In a study by Ziemkiewicz and Kosara [18], respondents reported that bubble [circle] charts seemed “unstable,” “uncontrolled,” and “disconcerting” and that the donut chart looked like it might “roll away.” Thus, circular chart types not only influence interpretation of data but also inform credibility [16]. Such connotation may unduly influence the overall impression of the subject matter, with less stable shapes inadvertently privileging items in the chart related to exerting greater control and force. Graphical models representing police UOF should not contain these inadvertent biases. 

**Crystal charts** organize hierarchical taxonomies, much like an organizational chart but in circular form [19]. A main concept sits at the centre and is divided into primary subconcepts in the first ring. Each subconcept is further subdivided in the second ring, which are further subdivided in the third (and so on). Some features of the Ontario UOF Model are permissible in crystal charts, including elements that cut across multiple rings (e.g., hard and soft physical control). However, the logic of a hierarchical taxonomy quickly breaks down, as there is no strict correspondence of subdivisions as the reader moves outward. At the centre of the Ontario UOF Model is the situation that the officers are in and is the core concept in police UOF decision-making. The officer’s assessment of the situation is broken down in the first ring, followed by suspect actions in the second ring, and the first of several force options in the third ring (now in colour). 

The challenges inherent in the use of **cycle charts** are reviewed in Section 3. 

### 2.2. Three-Dimensional Design Elements 

Three-dimensional pie and donut charts are more cognitively demanding, especially when including raised edges, shadows, and highlights. As Evergreen [16] reviews, “three-dimensional data displays slow down interpretation and often lead to inaccurate comprehension” (pp. 14–15). The edges of coloured areas in the Ontario UOF Model are beveled, perceptually reinforcing the discreteness of each element, rather than suggesting that it forms part of an evolving and dynamic process. The tilt of the circle in three-dimensional space distorts the size (and symbolic significance) of information on the graphic: portions at the top of the graphic appear farther away and smaller, while those at the bottom appear larger, which is not the communicative intent of the authors. The same can be said about distortions to text that are in the lower portions of the three-dimensional (3D) effect [17] (p. 64). Removing 3D effects makes chart elements appear visually precise [20] (p. 76). Collectively, ornamental embellishments simply add “visual noise” to the chart, that is, distracting details with no informational value (see Section 8—**Clarity**).

## 3. Cycle Models: The BC-CID Model

**Figure 3:**
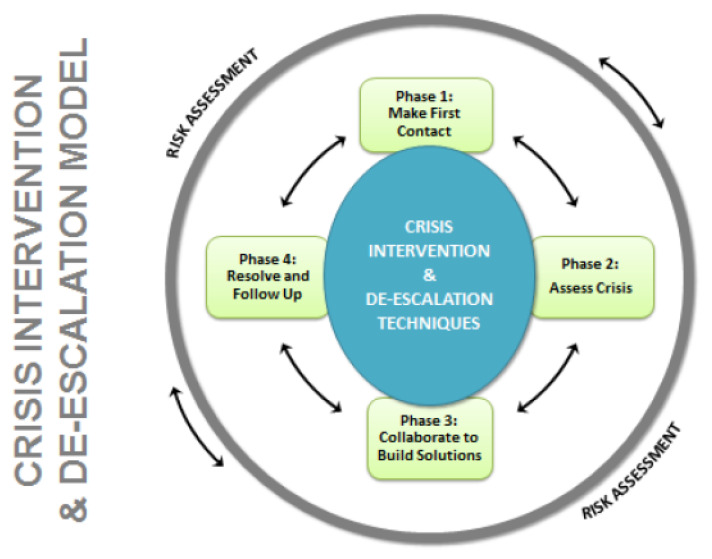
BC-CID Model. From [6] (p. 34) with permission from The Office of the Ombudsman of Ontario.

Cycle models show a recurring sequence of steps, stages, or phases. They are ideal for showing discrete activities that happen over time in a fixed order. The Ontario UOF Model contains a unidirectional cycle at its centre (i.e., arrows go one way). The British Columbia Crisis Intervention and De-escalation (BC-CID) Model (Figure 3) was reviewed by Ombudsman Dubé [6] as an alternative police UOF decision-making model. The BC-CID Model shows a bidirectional cycle (i.e., arrows go both ways), implying that the officer may return to earlier phases in accordance with “the natural ebb and flow of communication in a crisis situation” (p. 31). However, the use of numerous unlabeled arrows can create “crap circles,” or cycles that overuse multiple different shapes, directional arrows, angles, and curvatures to clarify an idea but end up obscuring it [9]. The BC-CID Model appears clear at first because of its simplicity. The model is simple only insofar as little information is presented and creates a false sense of understandability. As stated by Cairo [21]: “Simplicity isn’t just about reduction. It can (and should) also be about augmentation” (p. 97). What does the BC-CID Model really tell us about police UOF decision-making or intervention in crisis situations? How is this model specific to police? The genericism of the labels suggests that this sequence could apply to any sort of analytic process; there are no subject- or occupation-specific distinctions. Therefore, cycle models such as the BC-CID Model tend to oversimplify the activities that take place at each stage of police UOF decision-making and crisis intervention.

Although the BC-CID Model is part of a larger training curriculum that encompasses both UOF decision-making and crisis intervention, the visual model itself brings no additional understanding to the way in which an officer actually responds to a person in crisis. Several typographical (Section 7.1) and linguistic features (Section 7.2) of the BC-CID Model are also problematic. The title is stated on the left side of the model and repeated in the middle. Together with the repetition of “risk assessment” around the outer circle, such redundancy means a lost opportunity to provide additional meaningful information. Further, the re-orientation of reading vertically versus horizontally presented text requires uncomfortable eye movements and may elicit a sense of frustration in the reader [22]. Using technocratic broad language (e.g., “build solutions”) does not help the public understand (CACP goal #1) or provide officers with options (CACP goal #2) or language to articulate (CACP goal #3) what actions they might take to effectively address a crisis. Further, these terms may be interpreted very differently by police services, officers within the same service, and the public. Using broad language is an abstraction of a solution without committing to any substantive instruction. As with other simplistic models, extensive secondary source documents are required to interpret them (see Section 9.3), defeating the purpose of a stand-alone visual model.

To reiterate the purpose of the current critical review, just because a model contains poorly placed visuals does not necessarily mean that the information taught in the larger training program, or that the attempted message conveyed by the organization responsible, is not good. The quality of any police training program or visual aid must be evaluated on its own merits, which is also severely lacking in the applied police literature. The current analysis concludes that the visual representation of the BC-CID Model does not convey transparent, meaningful messages about occupationally relevant information surrounding UOF decision-making, crisis intervention, or de-escalation for the police or the public (CACP goal #1).

## 4. Staircase Models: The Las Vegas Model

**Figure 4:**
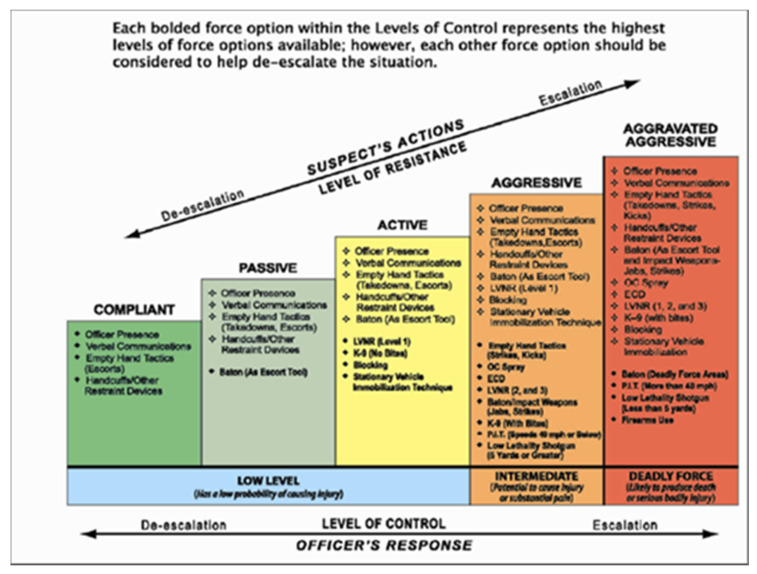
The Las Vegas Model. From [6] (p. 35) with permission from The Office of the Ombudsman of Ontario. All available versions of the Las Vegas Model are of poor resolution, limiting the clarity and interpretability of the presented text.

In contrast to the circular model types reviewed above, police UOF decision-making is often represented in the form of linear continuum models, or staircase models (Figure 4). Staircase models are intended to provide a reflective approach to selecting the least intrusive force option from a list or “menu” [23]. Also represented in the Ontario UOF Model, force options typically begin with the officer’s presence or verbal commands. Force options progress with increasing intrusiveness or potential for bodily or deadly harm, ending with lethal force. The accompanying language suggests deliberation or critical assessment, of which the force option is the most appropriate (i.e., “escalation,” “de-escalation”), with the goal of every potentially violent encounter being successful de-escalation. Both language and other visual features, including directional arrows, suggest that officers can consider progressing up or down the UOF continuum. While progressing through every force option may not be appropriate in all situations, a visual continuum implies a sequential approach [23]. Similar to the analog clock metaphor of circular models, the staircase metaphor combined with colours leads the eye (see Section 6), especially for readers of left-to-right languages, and connotes moving from one state to another. The resulting interpretation is that escalation is an inevitable outcome. The designers of the Las Vegas Model (Figure 4) have attempted to counter this inevitability by adding multiple visual elements that only confuse interpretation of the information present. Specifically, there is a juxtaposition in that moving up the top directional arrow (or escalation) is dictated by subject behaviour and moving down (or de-escalation) the bottom arrow is the goal of officer behaviour, which is not what stairs visually imply (left-to-right movement toward escalation). Labeling the same axis twice (i.e., suspect’s action/level of resistance, level of control/officer’s response) is redundant, confusing, and not scientifically supported [17] (p. 61).

Staircase models often make heavy and problematic use of text to express the numerous UOF options available to officers in a broad range of encounters that vary from no threat to life threat (see Section 7). As reviewed in Section 8, the use of numerous visual features, including text, colour, and directional arrows, reduces their interpretability and clarity. The Las Vegas Model specifically features a significant number of bullet points and acronyms, which are not fully articulated thoughts and violate the *principle of portability:* all information necessary for interpretation of a graphic should be contained within the graphic itself, unless there is a reasonable expectation that the audience will already know a particular item (see Section 9.1—Accessibility). When incomplete thoughts and acronyms are used, observers are required to scan through the entire chart to find explanations or search through a separate source document (see Section 9.3). Both of these options require significant effort and are unlikely to be undertaken unless this information is explicitly provided as during formal training. Source documentation has been separated from the police UOF models included in the current review, and therefore they cannot stand alone.

### Non-Linearity of Police UOF Decision-Making

Both police stakeholders and academic researchers alike have criticized the use of staircase models, suggesting they fail to serve officers’ needs during time-pressured encounters [24,25,26]. The CACP [4] UOF model framework discourages linear representations of force options, such that these models can appear rigid and prescriptive (i.e., officers must exhaust all UOF options at one level before considering alternative options). Linear continuums may be superficially attractive, especially when coupled with a euphonic acronym that purports to make the steps and options easier to remember. However, such implicit encoding is problematic and has been suggested by expert law enforcement instructors to result in “guaranteed hesitation in the face of a threat,” especially when an officer is faced with applying higher or lethal force [23] (p. 29). The CACP framework also criticizes linear continuum models for failing to accurately reflect the dynamic nature of potentially violent encounters. Suspect behaviours and appropriate force options are visually represented in discrete categories but are much more ambiguous in real-world contexts. The officers’ perceived level of threat and reasonable force options are continuously assessed and updated by ongoing changes to the environment and subject. Situational changes may not progress in sequence (i.e., compliant to posing a lethal threat), and officers may arrive to a scene already at the highest level of threat. The dynamism and complexity of police UOF decision-making are inadequately represented in linear continuum models and difficult to capture in any static graphical format.

Insights from cognitive neuroscience also suggest that the stepwise processes outlined in linear continuum models are not intuitive because the brain does not process information related to decision-making and action in a linear stepwise fashion [27,28,29]. By mentally progressing through a linear continuum of force options, officers squander precious time and cognitive resources. As a result, officers are at increased risk of reacting to a suspect’s behaviours, which is invariably slower than acting with purpose [30]. Officers have also reported using intuitive rather than analytic decision-making strategies under high-pressure contexts as a result of induced perceptual, cognitive, and physiological impairments [31]. Being able to accurately and quickly assess and choose the most appropriate force option for any given situation is indeed the central goal of police UOF training [3]. Therefore, pedagogical approaches that train officers in making UOF decisions in a linear (i.e., slow, analytical) fashion may put them at risk (see [32]). Therefore, increased complexity of UOF decision-making models may compromise learning outcomes in the absence of empirical evaluation of their effectiveness in conveying intended information.

The following sections of this review will critically evaluate the visual features common to all three chart types reviewed above, including colour, luminance, and saturation (Section 5); implied and visually cued motion (Section 6); text, language, and abstraction (Section 7); and clarity (Section 8). Insight into the practical challenges of developing, designing, and implementing effective graphical models of police UOF decision-making is given in Section 9, including accessibility, sizing, and graphical formatting and secondary documentation. To assist police practitioners, instructors, and curriculum developers and designers, we conclude our review with evidence-based recommendations for designing visually effective police UOF decision-making models (Section 10).

## 5. Colour, Luminance, and Saturation

The colour palettes used most commonly in police UOF models, including the Ontario (Figure 1) and Las Vegas (Figure 4) Models, feature basic primary (i.e., red, yellow, blue) and secondary (i.e., green, orange) colours. Colours are also typically presented on a progressive scale, with red reflecting the greatest level of threat or UOF response. Although these colour palettes may be a default in common processing software (e.g., Microsoft Office), the problem with using this kind of rainbow is that it gives emphasis to certain colours while also deemphasizing others. Theorized to be a product of evolutionary natural selection, red is the most perceptually salient colour to the human eye among individuals with unimpaired trichromatic colour vision [33,34]. Therefore, graphical elements intended to capture immediate attention are typically assigned the colour red, similar to highly salient objects in real life (e.g., stop signs and traffic signals, fire alarms, siren lights). Consistent with police UOF training practice, it is likely that designers of the Ontario and Las Vegas Models intentionally assigned lethal force the colour red to reflect the highest level of threat or danger. Red cues have also been shown to enhance the force and velocity of motor outputs [35], perhaps facilitating and reinforcing the “colour-force option” associations in police UOF models. However, red colour assignment to lethal force options may unintentionally de-emphasize other elements, including lesser force options (e.g., tactical considerations and communication). From a public communication perspective, highlighting lethal force is not consistent with the relatively low proportion of total police-public encounters that result in a use of lethal force [36] (see Section 8.2). Therefore, the use of colour, and particularly red, has represented lethal force as the most prominent feature of common police UOF decision-making models.

**Figure 5:**
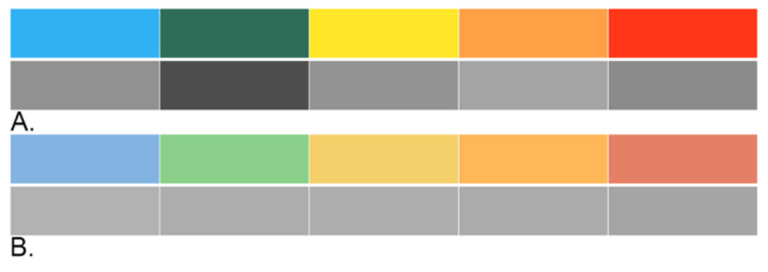
Colour saturation and luminance. Comparison of a high-saturation colour palette and the corresponding greyscale values (**A**) with a low-saturation colour palette and corresponding greyscale values (**B**).

The presentation of adjacent colours produces high levels of luminance contrast, which are perceived as borders or divisions. Luminance refers to the objective measure of light reflected off a surface, providing a range of values between black and white [37] (p. 80). Colour and luminance value contrast (i.e., area of greatest perceptual difference) are “pre-attentive” features that are processed “at a glance” by the viewer, prior to conscious attention [37,38,39]. Saturation refers to how “pure” a colour is perceived to be by the viewer. We describe high-saturation colours as “vivid” or “intense” [37] (p. 118). Colours at full saturation are not of equivalent luminance values, as illustrated in Figure 5. Therefore, luminance contrast increases the salience of some colours (e.g., red, green) while comparatively reducing the salience of other colours (e.g., orange, blue). Contributing to unequal salience is the chromostereoptic effect induced by adjacent contrasting colour segments. Chromostereopsis refers to a visual illusion whereby one colour appears to advance and the other appears to recede when they are in proximity to one another on a two-dimensional plane. This occurs primarily with the red-blue colour combination, as seen in the Ontario UOF Model (Figure 1). For most viewers, the proximity of these two highly saturated colours will give rise to an illusory effect where the red segment appears to be closer to the viewer than the blue segment, drawing greater attention to the red colour [37]. Effective graphics should limit highly saturated colours to very small areas that demand focus and use low saturation colours in larger areas where general readability is the priority [40] (Figure 5).

An alternative to colourful representations of progressive scales or continuums is the use of greyscale, as in the centre ring of the Ontario Use of Force Model (Figure 1). One problematic issue with the continuum used here is that people are being asked to judge a taxonomy of unfamiliar concepts (cooperative behaviour to serious bodily harm or death) superimposed on a greyscale continuum indicating categories that typically would have finite definitions and boundaries. The unfamiliar concepts, together with their placement on top of a shaded gradient, significantly increases the reader’s cognitive load. Although all graphics will increase cognitive load, attentional resources are finite and highly complex graphics can quickly induce fatigue [17]. Effective visual designers must consider the cognitive load being placed on the reader and reduce extraneous details that distract from essential information [11,12,41], including the combination of greyscale and colour features as observed in the Ontario UOF Model. The contrasting visual treatment of assessment (rendered in greyscale) from the surrounding UOF options (rendered in highly saturated colour) creates a striking visual distinction between these two components of the model. Moreover, the low-level contrast between text labels and lighter areas of the gradient (e.g., see the “passive resistant” text label), reduces the readability of this central graphic element. 

Together, these colour, luminance, and saturation features serve to **emphasize** lethal force options while **de-emphasizing** lesser force options (including de-escalation) and the continuous processes of situational awareness and assessment that are crucial for appropriate police UOF decision-making [3]. 

## 6. Implied and Visually Cued Motion

In addition to reflecting a non-linear progression of force options, stakeholders and practitioners have indicated the following important design criteria for police UOF models [6]:

- The model should promote continuous critical assessment and evaluation of each situation.
- The process should be seen as dynamic and constantly evolving.

The police UOF models reviewed presently employ several design features to portray “continuous,” “dynamic,” and “evolving” processes in static graphical form. Situational or risk assessment is explicitly written in the circular models (Ontario, BC-CID), but at opposing locations of the circle (centre versus outer ring). Differences in visual treatment (i.e., colour versus black and white) create a perceptual, and therefore conceptual, separation between assessment and action. Directional arrows used in all of the reviewed models can be an effective cueing strategy that guides the eye [42] and in circular models suggest that situational and risk assessment are a continuous, looping cycle. However, this is contradicted by the use of a white-to-black gradient overlaid with text labels to identify increasing levels of subject aggression. The gradient employs a subtle shift in value that is an effective method for communicating the wide continuum of behaviours ranging from “cooperative” to the threat of “serious bodily harm or death.” Yet, levels of increased aggression are made more visually salient by the high level of contrast between the white label and the black gradient beneath. While the inner wheel may effectively depict the process of continuous assessment, it fails insofar as it creates a cycle that visually escalates and is visually isolated from the corresponding outer UOF options [5]. In staircase models, bidirectional arrows may imply continuous assessment, which allows for moving back and forth between lower and higher levels of force. However, arrows visually reinforce the linear and progressive representation of suspect aggression and officer force responses from low to high.

**Figure 6:**
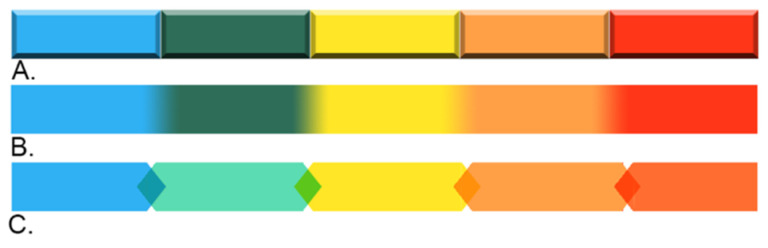
Visual representations of a dynamic overlapping process. (**A**) 3D form, (**B**) colour gradient, and (**C**) overlapping arrows.

With respect to the second design criterion, of reflecting a dynamic and constantly evolving process, the use of progressive colour scales (green, yellow, amber, red) on the Ontario (Figure 1) and Las Vegas (Figure 4) Models is suggestive of incremental forward motion, which is not conducive to backward movement through the cycle (i.e., de-escalation). While the choice of these colours is appropriate for communicating increasing levels of threat and response, this representational system also suggests the progressive sequence of traffic lights—another familiar convention that, like the clock, guides the eye in a specific direction. In the case of circular models, the eye is guided in a clockwise direction, while the staircase model guides the eye in a linear left-to-right direction. Specific to the Ontario UOF Model (Figure 1), the outward extension of colour segments is also confusing. While it is meant to suggest that there is an overlap between force options and that multiple options may be used at once [6], this information is not effectively conveyed from a graphical design perspective due to inconsistencies in visual language. The inner ring describing assessment represents dynamism using arrows, while the outer ring represents dynamism using a combination of area and colour. Overlap and bidirectional transitioning between force options might be represented more effectively with the use of gradients or arrows (Figure 6).

Given the combination of visual features that prompt specific eye movements, the circular and staircase UOF models reviewed presently do not clearly communicate the continuous and dynamic nature of the assessment process. Viewers are more likely to read the force options presented as a linear progression rather than as a fluid continuum of evaluation.

## 7. Text, Language, and Abstraction

The following sections will critically appraise the use of text (i.e., font, typeface), language, and abstraction to convey meaning in graphic visual models, including their impact on visual and cognitive information processing. 

### 7.1. Typography

As with the use of colour, the design of text labels can strongly influence the way that visual information is encoded by the viewer. Typefaces, or fonts, should be selected with the goal of preserving high levels of legibility and readability. Legibility means that the individual letter shapes are easy to perceive without eye strain or heavy concentration. Readability means that words and blocks of text are easy to read and are aesthetically appealing. Part of that aesthetic appeal comes from text that gives off a crisp and clean impression; that is, is tidy and uncrowded in appearance. Typeface rendered in upper case (i.e., all-capital letters) makes it difficult to quickly scan and read labels. When text is set in upper case, readability performance is impeded, with reading speed slowed by as much as 20 percent [43]. Reading can be further impeded by tight tracking (space between individual letters), making it more difficult to distinguish between the individual letter forms. Reading is optimal when uppercase and lowercase letters are used in combination, as in the content of the BC-CID (Figure 3) and Las Vegas (Figure 4) Models, as well as the legends of the Ontario (Figure 1) and Las Vegas Models. Words have a distinctive shape formed by variation in ascending and descending characters, a feature that is lost when words are written using all-uppercase characters (Figure 7). 

To further promote legibility and readability, professional typefaces offer designers the flexibility to tune the type given space constraints (i.e., for different sized graphics, see Section 9) using a wider range of heights, widths, and weights (i.e., not just regular and bold but in-between weights too). The bold typeface used to convey the highest-force options at each level of control in the Las Vegas Model (Figure 4) visually captures attention but may also mislead interpretation of these options as the most common or appropriate, especially if readers ignore the top header of the graphic. Multiple changes in reading direction (i.e., left-to-right, top-to-bottom, diagonally or circular forward and backward, central and peripheral) exert a significant amount of strain on the oculomotor muscles and can quickly induce fatigue. For further details about typographic best practices, see [22,44,45].

### 7.2. Abstract Language and Jargon

In addition to typographical considerations, the text labels used in most police UOF decision-making models lack clarity by making use of abstract and inconsistent language. The models reviewed in the current work violate many of the *principles of plain language* necessary for clear and meaningful visual communication. The following are some examples of these violations, with the latter two especially relevant for public communication of police UOF decision-making (CACP goal #1):

- Noun-ifying verbs, verb-ifying nouns, and adjective-izing action words or alternately using verbs, adjectives, and nouns as modifiers for describing subject behaviour (Ontario UOF Model);
- “Zombie words” or “dead words” [46] that have many possible meanings due to their overuse and lack of specificity (e.g., “resources,” “resolve,” “assess”) and cause confusion by obscuring specific criteria or conveying unrealistic connotations (e.g., the use of terms “assaultive” and “aggressive” makes it unclear what specific activity is generating a sense of threat);
- Trash-can categories that group together a miscellany of left-over items (e.g., “other”);
- The use of euphemism, loaded terms, vagueness, and idioms, which are all forms of undue political evasion intended to obscure reality and avoid accountability (e.g., “soft control,” “build solutions”) or are turns of phrase that are not easily interpreted by those who speak English as a second language (“active resistant”);
- Unclarified jargon, undefined acronyms, or undefined terms that are of a technical nature, leaving the audience unclear as to meaning (e.g., “tactical considerations,” “intermediate weapons,” “ECD,” “PLT,” “LVNR”);
- Unexplained complex concepts, or umbrella terms that summarize two or more complicated concepts, which are problematic because of lack of specificity (e.g., “de-escalation,” “crisis intervention”).

Overly broad categories without definitions are at the risk of being interpreted differently across police services and the public, especially without the use of a secondary source document (Section 9.3). For example, broad category terms like “tactical considerations,” “communication,” and “perception” may mean different things to different officers (e.g., frontline versus special tactical unit) and to the public. There are limits to what can be conveyed by a model that is simply an arrangement of basic shapes containing general text labels. Basic shapes (circles, squares, arrows) do not evoke vivid images in the imagination. They are merely placeholders in the spatial organization of information. When meaning is overly reliant on broad, generic, vague, or abstract labels, it lends itself to a variety of interpretations and makes it harder to form mental images of intended information. Previous research demonstrates a decrease in cognitive load during visual imagery relative to observational information processing [47]. Greater availability in cognitive resources allows individuals to learn and remember more and facilitates understanding of complex processes [16] (pp. 18–23). 

The models reviewed are further subject to a high risk of misinterpretation due to the compounding of multiple abstractions. When substantive meaning is low, the potential for misinformation is high. Assuming that implicit information known to oneself is self-evident to others is known as an “abstraction trap” or a “knowledge trap” [13]. Vague representations of complex concepts often fall victim to knowledge traps insofar as it becomes harder to communicate ideas with precision and specificity. For example, individuals may have difficultly interpreting or remembering the various (abstract) force options presented because of abstractions used to describe an officer’s assessment of their level of control (e.g., an officer is present in a situation, applies tactical considerations based on their perception, uses communication, then applies soft physical control because the subject was passively resistant). Moreover, general labels can be treated with a “concreteness” that is not justified given their role as summarizing devices. The solution to knowledge traps is to reintroduce visuals and descriptive text to clarify the subject matter. 

Together, the lack of visual images and high number of abstractions representing the complexity of police UOF decision-making in the presently reviewed models may lead to errors in interpretation and at worst in their application.

## 8. Clarity

As a central design principle, clarity of visual information can promote knowledge translation. The following considerations can help visual model developers to maximize clarity while also avoiding misrepresentation of chart information by considering proportionality of key design features.

### 8.1. Chartjunk and Data-to-Ink Ratio

The overwhelming amount of visual design features in the currently reviewed police UOF models violates the important design principle of clarity. Edward Tufte, a pioneer in the field of information design, identified two concepts relevant to the current analysis: the “data-to-ink ratio” and “chartjunk” [10,11,12]. The “data” in the former concept refers to the visual elements that describe the data or information directly; this is compared with the “ink,” or extraneous visual elements, contained within the display in its entirety. Chartjunk refers to unnecessary “ink” and embellishments that hinder communication of “data” [17,48] and is not recommended. A common mistake on the part of graphic model designers is to include decorative chartjunk with the belief or intention that they make the model appear more advanced, complex, or engaging. This belief is then used to defend, and even promote, the use of unnecessary design components as essential to the message designers wish to convey [10] (pp. 107–122). 

In the Ontario UOF Model (Figure 1), the use of 3D and shadow effects, pixelated bitmap quality graphics, overuse of multiple chart types, colours, and shapes are but some of the components considered “noise” or chartjunk, meaning that they do not aid in understanding the model. The reliance on slight indented lines, shading, and colours to communicate differences send a confusing message of which visual information is to be relied upon to make decisions about the information presented. Visual noise is also induced by the use of highly saturated colours represented over large areas adjacent to one another [40] (Figure 1 and Figure 4). The perceptual interference of competing colours can also result in eye fatigue [49], further reducing the effectiveness of the graphic in conveying information. The overwhelming use of bullet points, evident in the Las Vegas Model (Figure 4), may be useful for listing all available and sanctioned force options as a police UOF training tool. However, the abundance of text forces any reader (novice, expert, or layperson) to juggle too many concepts in their minds at once and becomes a “word salad.” The relationship between concepts in word salads are not defined or lost, as terms become amalgams that require the reader to piece them together and imbue their own meaning. This makes the relationships between terms and criteria (i.e., levels of aggression or control) even more ambiguous and increases the likelihood of misunderstanding the model.

The ill effects of chartjunk go beyond simple confusion; Tufte warns that infographics high in chartjunk preclude observers from asking more important questions regarding the quality of data, analysis, and evidence being presented [10]. These concerns coincide with the sentiments reported in stakeholder reports and recent inquests regarding the quality of police UOF models in fulfilling their intended goals [5,6,7,50,51]. Together with the abundance of media coverage and conflation of UOF statistics across jurisdictions (e.g., large urban centres in the United States versus rural Canadian regions), publicly available police UOF models high in chartjunk may contribute to misrepresentation of the frequency of police UOF (see Section 8.2). Highly complex graphics may result in a greater reliance on materials that are easier to understand but not necessarily accurate or correct (e.g., erroneous media reports from other jurisdictions or social media). It is not only the graphical flourishes that dissuade consumers from attempting to understand a graphic but also the *perceived difficulty* of attempting to learn the information presented. Graphics with a low data-to-ink ratio encourage observers (including police) to read on, explore the information, learn new information, and even re-educate themselves to confront possible biases or assumptions [17]. 

### 8.2. Proportionality

Researchers examining how the brain processes risk and threat report that unrealistic representations of threat frequency inadvertently primes the belief that UOF encounters are more frequent than they actually are, and that force is used more often than it actually is [52]. Compounding of several visual features in the models reviewed above contributes to significant misjudgment of the actual frequency of UOF incidents by police, especially in Canada. In addition to limiting police decision-making to UOF options (i.e., excluding guidance on police decision-making for encounters not involving a UOF), the Ontario UOF Model and the Las Vegas Model dramatically overestimate the role and use of lethal force in police encounters. In a review of 10.9 million police-public interactions between January 2012 and December 2015, approximately 1 in every 1210 (or 0.0008%) calls for service to a large Canadian law enforcement agency involved a use of force incident [36]. Twenty-four of the 51 police services in Ontario, Canada, provided UOF reports from 2017, which indicated 6922 use of force incidents from 4,025,169 calls for service (approx. 1 in every 581, or 0.2%) [53].

Using chart segments clockwise or from left to right, from smallest to largest (or less to more), gives the impression that the very last segment is the most frequent and important and is generally discouraged [54] (p. 74). In the Ontario UOF Model (Figure 1), “serious bodily harm or death” is in the last quadrant of the inner greyscale circle, suggesting that it is the most frequently occurring type of encounter. In the Las Vegas Model (Figure 4), deadly force options appear to be the most frequent, as reflected by the tallest bar in the chart. On both of these continua, death is given the most visual emphasis through the use of colour and positioning, with passive or cooperative outcomes getting the least emphasis. These emphatic features directly support the ombudsman’s [6] critique that police UOF options rather than de-escalation options are being highlighted in current police UOF models. Together with chartjunk, disproportionate visual representation of lethal force on a continuum of all possible force options can contribute to public misperception (violating CACP goal #1).

## 9. Practical Challenges to Developing Static Visual Models for Police UOF 

To effectively convey visual information to a broad audience, good graphic models need to consider accessibility needs, including how sizing and graphic format can possibly distort final printing or presentation. Supplementing visual graphics intended for instructional purposes with secondary documentation is also critically discussed. 

### 9.1. Accessibility

All of the police UOF models reviewed in the current work are presented in colour, which immediately raises concerns regarding accessibility. It is estimated that about 1 in 12 people have a colour vision deficiency of some kind [20] (p. 136), with approximately 8% of males and 0.5% of females of Northern European ancestry experiencing red-green colour blindness [55,56]. Thus, conveying meaning by the use of colours in a graphic places a portion of the population at a disadvantage from an information visualization perspective due to visual disability. Accessible design features include the use of black-and-white imagery, high-legibility typefaces, clear type hierarchies, and high-contrast imagery with hard edges to aide those with low visual acuity. Materials for broad public communications should assume readers have an elementary school level of literacy [57,58]. Various resources have been developed to promote the effective use of plain language [59], including legislated use of plain language by all U.S. federal government agency communications [60]. Use of text explanation in full sentences also makes the graphic accessible to those who use text-to-speech technology [56] (pp. 61–67). 

### 9.2. Sizing and Graphic Format

Effective graphics should be designed to be legible and readable when printed on a standard 8½- by 11-inch page (in landscape mode at 100% page scaling). The use of vector graphics allows the graphic to be shown in larger sizes with no loss in quality. Currently reviewed graphics are bitmap images, meaning that each image is a matrix of small pixels at a fixed resolution, just like a digital photograph. A weakness of this graphic type is that the size cannot be adjusted without causing degradation of the image. The already blurred text of the Las Vegas Model (Figure 4) further exacerbates the issue of graphic format. In contrast, a vector graphic is composed of relatively positioned points, lines, and curves that are expressed as mathematical equations. This feature allows the graphic to be enlarged or shrunk to an arbitrary size without any degradation. In other words, it still looks crisp and clean (not pixelated or blurry) regardless of how large it is printed (notwithstanding any limitations of the particular printing device used). Vector graphics will also appear crisp and clean on the screen of any modern computer.

Consideration of graphic format in police UOF model design is important because these graphics are being reproduced in poster sizes (approx. 16 × 24 inches) and placed throughout police stations and training academies. In their current formats, posters of the reviewed UOF models are at risk of appearing blurry or pixelated and show signs of “ghosting” (i.e., subtle patterns around high-contrast edges caused by bitmap-file compression algorithms that become very noticeable at large sizes) [16].

### 9.3. Secondary Documentation

While visual models are necessary for training and representing standards of practice, they are not a substitute for complete lesson plans and skill-based training. It is also practically impossible to capture the infinite number of possible combinations of suspect behaviour and officer response outcomes in a single model, including the representation of decision-making that does not involve a UOF. Reviewing the essential components of, or processes involved in, police UOF decision-making lies beyond the scope of the current review; see [3,31,52,61]. Nonetheless, each model reviewed above attempts to capture essential aspects of police UOF decision-making, including situational elements (e.g., environment, individuals), implicit cognitive processes (e.g., perception, understanding, prediction), and the variety of force options available at varying levels of threat or danger. Relevant for both police training and public communication purposes (CACP goal #1), most police UOF models require secondary documents to define and describe included terms and concepts. Secondary documentation also provides insight into when and how police are intended and instructed to use the model(s) to make appropriate decisions during encounters with the public. That visual training models are not independently interpretable without the secondary source document is highly problematic, especially considering that such documentation is often not easily accessible or publicly available. To promote transparency and accountability of current police UOF practices to the public [6], model designers are encouraged to reduce the need for secondary documentation by creating stand-alone graphics or provide easily available links to such documentation. 

## 10. Recommendations for Evidence-Based Design of Visual Models for Police UOF Decision-Making

Police have increasingly promoted and adopted evidence-based practices [62], including collaborations with vision science researchers. For instance, recent investigations have used pupil, gaze, and fixation tracking during live-action lethal force scenarios to better understand police perception, attention, and decision-making; e.g., [63,64,65]. Similarly, the current critical review stands to provide evidence-based recommendations rooted in vision science and information visualization that can aid in the development of effective police UOF models that fulfill the goals defined by police stakeholders [4].

- Police UOF decision-making is a complex process that requires graphical models that display this complexity without being cluttered, overwhelming, or confusing. The content should be explanatory and purposeful [10,11,12]. Therefore, redundancy and repetition should be avoided to save visual space for informative and meaningful content.
- Models should represent UOF decision-making as a continuous and dynamic process that *emphasizes* backward motion (i.e., de-escalation).
  ○ Caveat 1: Donut charts that feature segments are not recommended because they require the observer to perform mental calculations and interpretations of the relative area occupied by each segment within the circle to gain some meaning from the information presented.
  ○ Caveat 2: Segmenting force options from least to most intrusive (i.e., clockwise or left to right, smallest to largest) suggests that the last segment (lethal force) is the most frequent and important [54]—therefore equally sized and represented force options are recommended to avoid misrepresentation and/or misinterpretation of the relative frequency of police lethal force responses.
- To reduce cognitive load, fatigue, and conflation of information, designers should use a single chart type and minimize the use of multiple different shapes, directional arrows, angles, and curvatures.
- Designers should avoid use of 3D effects to maintain visual precision of chart elements.
- If colour is used, designers should use colour palettes that are accessible to individuals with colour blindness; see [66].
- Highly saturated colours should be limited to small areas to draw visual focus, while low-saturation colours should be used in larger areas to promote readability [40].
- Use of greyscale, especially underneath imposed text, is not recommended because greyscales are prone to degradation under certain circumstances (i.e., photocopying, printing, different screen resolutions).
- Black-and-white (BW) graphics are recommended for several reasons:
  ○ They can be reproduced on a wider range of office equipment and at a reduced cost relative to colour graphics.
  ○ BW images and text have the highest contrast and therefore the best visibility and greatest portability, especially for viewers with visual impairment, including but not limited to colour blindness [20].
  ○ BW images avoid controversy related to the potential social or cultural connotations associated with particular colours.
- Visual features should be prepared with professional typefaces in vector graphical formatting. Additional shapes, boxes, and arrows should also be prepared to appear professional and clean to minimize appearing amateurish.
- For public communication purposes, each type of force encounter should be represented proportionately to its occurrence based on available UOF data or a statement referring to the proportion of each type of encounter should be featured on the public version of the model.
- Any new visual models representing police UOF, either for police training or for public communication purposes, should be empirically evaluated for their effectiveness in conveying information through scientific inquiry (e.g., focus groups, evaluation of learning outcomes compared to training with other visual UOF models).

## 11. Conclusions 

To meet the growing public outcry surrounding police use of force, numerous political and organizational groups have called for the investigation and/or reform of existing policies and practices [5,6,7,50,51,53]. The visual UOF models reviewed in the current work were either recommended to be updated (Ontario UOF Model) or suggested as replacements to current training and public communication materials (BC-CID and Las Vegas Models) [6,7]. To date, there has been no empirical evaluation of whether currently used models align with established frameworks [4] for communicating UOF decision-making to police or the public. Synthesizing perspectives from visual (graphic design, information visualization, visual neuroscience) and applied (policing) sciences, the current review sought to critically analyze key visual features of publicly available police UOF models that directly influence knowledge dissemination. In most cases, police are trained to respond adaptively to dynamic encounters with a variety of force and non-force options (i.e., verbal communication, non-verbal body language, positioning), with the goal of de-escalating and resolving critical incidents. However, the concurrent use of numerous visual features leads to disproportionate representations of lethal or deadly force encounters and inappropriately conveys linear, progressive, and prescriptive decision-making. There are challenges to visually (i.e., explicitly) representing complex and implicit knowledge related to UOF decision-making required by police officers in a static model. Practical design challenges are further complicated by the lack of universal definitions of and standards for police UOF training [3]. Nonetheless, the insights and recommendations provided in this critical review can contribute meaningfully to the development of evidence-based training and public communication tools for police UOF decision-making. 

## Figures and Tables

**Figure 7:**
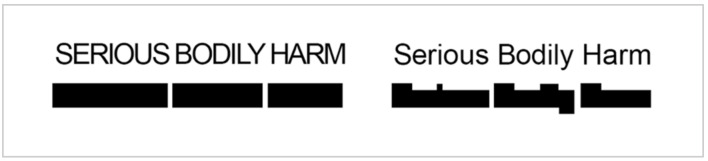
Typeface. Uppercase text forms visually uniform blocks that do not lend themselves to rapid discrimination. Words rendered in lowercase form distinctive shapes and patterns that are more quickly understood by the reader.

## Data Availability

No new data were created or analyzed in this study. Data sharing is not applicable to this article.
